# The Impact of ADH1B Alleles and Educational Status on Levels and Modes of Alcohol Consumption in Russian Male Individuals

**Published:** 2013

**Authors:** S.A. Borinskaya, A.A. Kim, A.V. Rubanovich, N.K. Yankovsky

**Affiliations:** Vavilov Institute of General Genetics, Russian Academy of Sciences, Gubkina Str. 3, Moscow, Russia, 119991; Moscow State University of Medicine and Dentistry, Delegatskaya Str. 20, Bld. 1, Moscow, Russia, 127473; Moscow Institute of Physics and Technology, Institutsky Lane 9, Dolgoprudny, Moscow oblast, Russia, 141700; Faculty of Biology, Lomonosov Moscow State University, Leninskie Gory 1, Bld. 12, Moscow, Russia, 119234

**Keywords:** alcohol consumption level, genetic polymorphisms, ADH1B gene, social factors

## Abstract

Alcohol abuse is one of the main reasons behind the low life span in Russia. Both social and genetic factors affect the alcohol consumption level. The genetic factors are alleles of the alcohol dehydrogenase ADH1B and aldehyde dehydrogenaseALDH2 genes. We have typed and found frequencies for the alleles in a cohort of 642 men, ethnic Russians. The individuals of the cohort were asked to complete a questionnaire in the framework of the Izhevsk Family Study (Leon et al., 2007, 2009) regarding the amount of alcohol consumed and on the type of hazardous alcohol consumption (nonbeverage alcohol consumption and the so-called “zapoï” which is a Russian term for a heavy drinking bout lasting for at least 2 days, when an individual is withdrawn from the normal social life). The ADH1B*48His allele was found among heterozygous individuals only (N=68, 10.6% of the cohort). The ALDH2*504Lys allele was also found among heterozygous individuals only (N=2, 0.3%) The effect of ADH1B alleles and the influence of the education level on the amount and type of alcohol consumed had not previously been studied in Russians. We have found that the amount of consumed alcohol is 21.6% lower (1733 g of ethanol per year) for ADH1B*48His allele carriers in the cohort of Russian men. The amount of consumed alcohol was found to be 9.8% lower (793 g of ethanol per year) in the case when individuals had a higher education as compared to those who had a secondary- or elementary school education level in the same cohort. Hence, the protective effect of the genetic factor (ADH1B*48His allele carriage) has proven to be more pronounced than the influence of the social factor (education level) at the individual level in the cohort of Russian men. Both factors have also proven to have a protective effect against hazardous types of alcohol consumption. Zapoï was not scored among individuals of the cohort with ADH1B*48His allele carriage (OR=12.6, P=0.006), as compared to 8.4% of “zapoï” individuals who did not carry the ADH1B*48His allele (genotype Arg/Arg).The percentage of individuals who consume non-beverage alcohol is lower (0.6%) in the subcohort of people with a higher education degree. This percentage is higher (6.0%, OR=10.0, P=0.004) in the subcohort of people without a higher education degree.

## INTRODUCTION

Alcohol abuse is admittedly one of the main causes behind the low life span in Russia. It is responsible, either directly or indirectly, for up to 60% of the deaths occurring to non-elderly males in Russian populations [Nemtsov, 2001; Andreev *et al*., 2008; Leon *et al*., 2009; Zaridze *et al*., 2009].

Exogenous ethanol is metabolized in humans predominantly by liver enzymes, alcohol dehydrogenase (ADH) and aldehyde dehydrogenase (ALDH), which are responsible for the sequential oxidation of up to 90% of consumed alcohol. Another alcohol oxidation route is driven by microsomal cytochrome P450 that converts about 9% of exogenous alcohol; peroxisomal catalase also has a minor contribution of about 1% [Ostrovsky, Sadovnik, 1984; Halej, Berndt, 1987; Luzhnikov, 1994].

Seven ADH genes characterized by distinct tissueand age-specific expression patterns have been identified in the human genome [Edenberg, 2000]. The first step of exogenous ethanol oxidation is accomplished predominantly by the *ADH1B*-encoded enzyme. A single nucleotide polymorphism in this gene corresponds to an *Arg48His *amino acid substitution that influences enzyme velocity, so that the histidine-containing isoform (corresponding to ADH1B*48His) is 100 times more active than the arginine-containing one (corresponding to ADH1B*48Arg) [Jornvall *et al*., 1984; Matsuo* et al*., 1989].

Acetaldehyde formed by ADH from ethanol is consequently oxidized to acetate by ALDH. Up to 95% of acetaldehyde is converted into acetate by mitochondrial ALDH encoded by *ALDH2 *[Goedde *et al*., 1987; Hsu* et al*., 1988]. A single nucleotide polymorphism in this gene corresponds to a *Glu504Lys *substitution, which yields an inactive protein in homozygous individuals. Moreover, since ALDH functions as a homotetramer with one dysfunctional subunit inactivating the entire complex, heterozygous persons possess only about 6% of the ALDH activity characteristic of *504Glu *homozygous humans [Crabb *et al*., 1989].

The observable toxic effect of consuming large amounts of exogenous alcohol is caused not by alcohol itself but by its primary metabolite, acetaldehyde [Halej, Berndt, 1987]. For ALDH2*504Lys carriers, considering his or her low acetaldehyde detoxication rate, a drinking bout may lead to high blood acetaldehyde levels. Hence, the toxic effects of alcohol consumption for these individuals are much more pronounced [Gelernter, 2009]. Therefore, heterozygous carriers of ALDH2*504Lys consume less alcohol and are at lower risk of alcohol dependence than those lacking this allele [Wall *et al*., 2000; Kim *et al*., 2008]. A similar protective effect, although less pronounced, was shown for the ADH1B*48His allele both in combination with ALDH2*504Lys for Japanese and Korean populations [Matsuo *et al*., 2006; Kim *et al*., 2008] and on its own for white Americans and Australians [Sherva *et al*., 2009; Macgregor *et al*., 2009]. For Russian populations, there is still no evidence that these alleles affect the levels of alcohol consumption.

The ALDH2*504Lys allele frequency in Asian populations varies from 40% in East Asia to less than 1–2% in Central Asia. The allele is virtually absent in European populations, and among all the examined Russians, only one individual was shown to be an heterozygous carrier of ALDH2*504Lys [Li *et al*., 2009]. The ADH1B*48His allele frequency is also very high for East Asia (70%). In Europe it varies between less than 1% and 8–10%. In Russia, ADH1B*48His carriers were shown to constitute from 5% to 15%, which corresponds to an allele frequency of 2.5–8% [Borinskaya *et al*., 2009].

In this study, we focused on specific alcohol consumption patterns characteristic of Russian males with ALDH2*504Lys and the ADH1B*48His alleles.

## MATERIALS AND METHODS

Blood samples of 642 Russian males aged 22 to 59 collected during the 2008–2009 Izhevsk Family Study program [Leon *et al*., 2007; Andreev *et al*., 2008] were used as material for this study. The probes were supplemented with biochemical and immunological assay data. The corresponding questionnaire forms (including the fields’ ethnicity, educational status, and alcohol consumption habits) were filled out by the individuals and their relatives under the supervision of competent personnel trained for the Izhevsk Family Study program [Leon *et al*., 2007; Andreev *et al*., 2008].

The participants were questioned on the amounts of consumed alcoholic beverages and how often they drank them. The individual annual volume of pure ethanol was inferred from data on the periodicity of intake, beverage volume, and alcohol content. The ethanol content of the beverages available in Izhevsk was taken as stated on labels. The alcohol content of the vodka sold in the city was measured independently using conventional laboratory techniques (see Table 1 for specific alcohol consumption characteristics).

**Table 1:** Alcohol consumption indices among Russian men in Izhevsk 2008-2009

Alcohol consumption	Number of individuals (%)
Total persons	642 (100%)
Abstainers (a year before the survey)	83 (12.9%)
- former consumers	80 (12.5%)
- lifelong abstainers	3 (0.5%)
Alcohol consumed weekly	322 (50.2%)
- including daily consumption	44 (8.9%)
Individuals had at least one *zapoï* episode during the past year	48 (7.5%)
Nonbeverage alcohol consumers*	30 (4.7%)

*Nonbeverage alcohol means an alcohol containing liquid not supposed to be used for drinking purpose (eau-de- Cologne, pharmaceutical alcohol containing tinctures, alcohol containing liquids used for technical needs, etc.).

Genomic DNA from the blood samples was purified with the QIAmp DNA Blood Mini Kit (QIAgen). The genotyping assays for ADH1B*Arg48His and ALDH2*Glu504Lys were based on the duplex fourprimer PCR design [Tamakoshi *et al*., 2003].

Descriptive statistics and a multiple regression analysis were performed using the STATISTICA 6.0 software. Intergroup variances were estimated by the non-parametric Mann-Whitney test. Odds ratio (*OR*) calculations and the Fisher’s exact test for significance were performed using the WinPepi program [available at www.brixtonhealth.com/pepi4windows.html; Abramson, 2004].

The contribution of the genotype (*D*) and other factors (*I*) to alcohol consumption was calculated according to the formula:

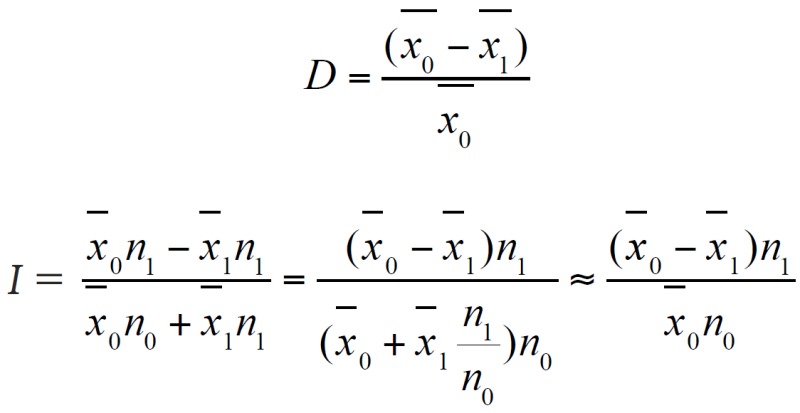



where *D *is the relative risk for Arg/Arg genotype carriers, –*x_i_*is the average level of alcohol consumptions for the *i *-genotype, *ni *is the number of *i*-genotype carriers, and *I *is the input of the factor influencing the level of alcohol consumption in the group.

## RESULTS AND DISCUSSION

The alcohol consumption indexes for the given sample are presented in Table 1. The average index of pure ethanol consumption is 6765±364 g per individual per year, excluding nonbeverage alcohol consumers. Surprisingly, it is approximately twice as low as the average pure ethanol consumption per individual in Russia [24]. The discrepancy can be explained by at least two factors: *i*) the share of heavy drinkers among young people in the group is rather low; *ii*) The amount of alcohol consumption depends on the type of registration in the questionnaire [25]. The amount of alcohol scored by the period type of registration (over a month) shows a twofold lower level of consumption as compared to the day-by-day type of registration for Russians [26].

**Fig. 1:**
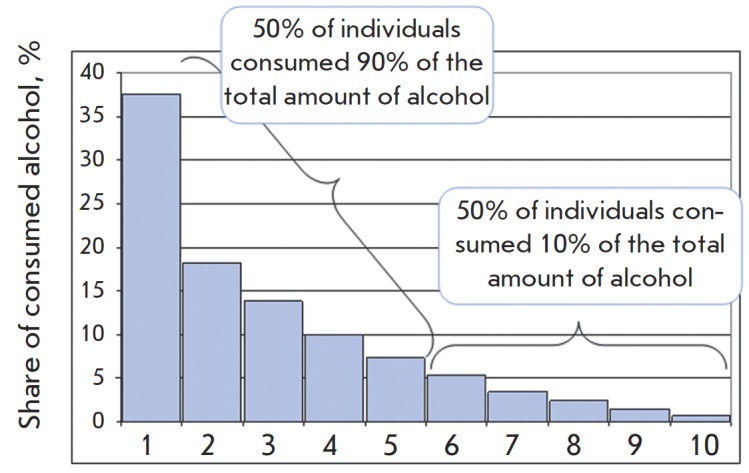
Share of consumed alcohol in 10 subgroups (53 men in each group) ranked by the alcohol consumption level. Abstainers are excluded; nonbeverage alcohol consumers are excluded

According to the questionnaire-based estimates, one half of the total amount of alcohol drunk is attributed to 14% of the sample. The distribution of individual consumption values (related to the integral sample consumption) in subgroups ranging by consumption level from the maximal to the minimal is presented in Fig.1.

All individuals in the sample were genotyped for the ADH1B*Arg48His and ALDH2*Glu504Lys polymorphisms. Heterozygous ADH1B*48His carriers constituted 10.6% of the sample; no homozygous carriers were identified (Table 2). The distribution of genotype frequencies corresponded to the Hardy-Weinberg equilibrium distribution. The total ADH1B*48His allele frequency in the sample (5.2%) corresponded to the data obtained for the previously studied Russian samples [Borinskaya *et al*., 2009].

**Table 2:** ADH1B*Arg48His allele and genotype frequencies

Genotype frequencies (Number of individuals)			Allele frequencies (SD)		c2 (p-value)
Arg/Arg	Arg/His	His/His	G	A	
0.894 (574)	0.106 (68)	0	0.947+0.008	0.053+0.008	2.01 (0.157)

Since only two individuals in the sample were identified as ALDH2*504Lys heterozygous carriers (0.16% allele frequency), the allele was eliminated from further analysis. The individuals who consumed nonbeverage alcohol were excluded from the analysis, since it was impossible to estimate the amount of pure alcohol consumed in that case.

The ADH1B*48His allele association with the level of alcohol consumption was analyzed using two approaches: between-group comparison of the allele frequency (ranged by consumption values); comparison of consumption values for ADH1B*48His and ADH1B*48Arg genotyped individuals in the sample stratified by age.

For the first approach, the total sample was subdivided into 4 groups (Table 3). One group included all nonbeverage alcohol consumers (30 individuals); another group included people who reported consuming no alcohol for at least a year (83 individuals). The remaining alcohol consumers (529 individuals) were ranked by the alcohol consumption level and subdivided into two nearly equal subgroups (Table 3): one having a higher consumption level (“heavy drinkers,” 264 individuals) and the other having a lower consumption level group (“moderate drinkers,” 265 individuals).

**Table 3:** ADH1B*48His carrier frequency and higher education level at different alcohol consumption levels

Alcohol consumption level and style	Total number of individuals	Mean alcohol consumption level (g of ethanol per person per year)	ADH1B*48His carrier frequency % (N)	High education level % (N)	
Higher level of alcohol consumption	264	13517	8.7% (23)	22.3% (60)
Lower level of alcohol consumption	265	2162	14.0% (37)	33.6% (89)
Abstainers	83	0	8.4% (7)	9.6% (8)
Nonbeverage alcohol consumers	30	Not determined	3.3% (1)	3.3% (1)
Total:	642	- -	10.6%	24.2% (158)
				
At least one zapoï episode during the past year	48	15984	0	18.8% (9)
No *zapoï* episodes during the past year	574	7278	11.8% (68)	24.4% (140)
Total	642			

Note. Number of individuals is given in parentheses.

The ADH1B*48His allele carriage frequency in the “moderate drinkers” subgroup was found to be 13.4% as compared to 8.7% in the “heavy drinkers” subgroup. The result does not contradict the hypothesis on the protective role of the ADH1B*48His allele against a high level of alcohol consumption; however, the result is not quite statistically valid (p=0.074, two-tail Fisher test).

Carriers of the ADH1B*48His allele in the “no alcohol consumption” group constituted 8.4%. As long as all but three individuals in this group indicated drinking in the past, a plausible cause of drinking cessation at least for part of this group could be related to health problems. This assumption is supported, in particular, by an increased occurrence of *Treponema pallidum *reactive antibodies in the blood samples of this group. The antibodies were detected in 4 of 83 individuals in the “no alcohol” group (4.8%) as compared to 3 of 264 “moderate drinkers” (1.1%) and 5 (1.9%) of 265 “heavy drinkers.” A more detailed analysis of individual reasons for the cessation could be of interest.

Only one carrier of the ADH1B*48His allele was identified (3.3%) among the non-beverage alcohol consumers.

The groups also differed by the educational status of individuals (Table 3). Among the total sample, 158 individuals (24.6%) possessed a degree in higher education. The percentage of individuals with higher education was 33.6% and 22.3% for “moderate drinkers” and “heavy drinkers,” respectively. This difference is statistically significant (OR=1.72, p=0.007 by two-tailed Fisher’s test); i.e., a degree in higher education is less frequently accompanied by heavy drinking habits as compared to lack of a degree.

In the abstainer group, the percentage of individuals with a degree in higher education was 9.6% of the group, which was less than in both previous groups (Table 3). This difference is statistically significant (p < 0.02). Taking into account the lower probability of heavy drinking for individuals with higher education, the smaller percentage of individuals having a degree in higher education in the “abstinent” group may be regarded as an indirect indication of excessive consumption in the past as one of the reasons for cessation. This consideration is also supported by weaker representation of the ADH1B*48His allele carriers in this group (8.4%) that is virtually equal to their representation in the “heavy drinkers” group (8.2%).

In the nonbeverage alcohol consumer group, only one out of 30 individuals (3.3%) declared having a degree in higher education.

**Fig. 2:**
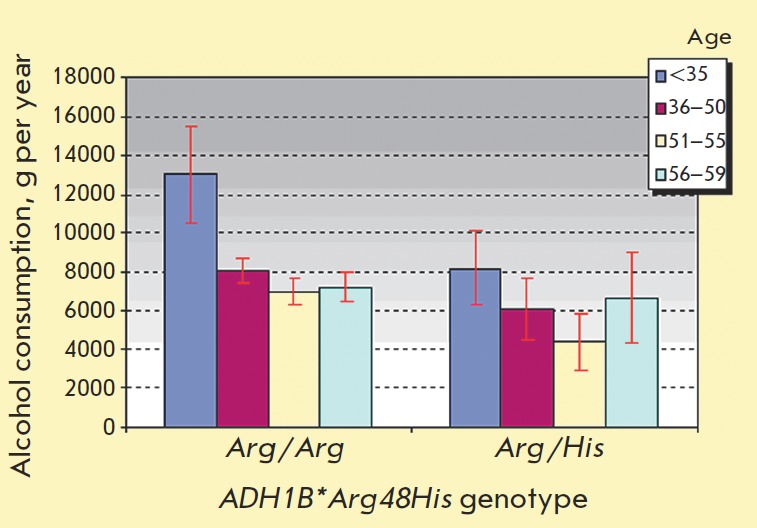
Mean alcohol consumption level (g per person per year) in age groups for Arg/Arg and Arg/His genotype carriers

Alcohol consumption levels with relation to genotype were determined for the total sample and also separately for different age groups. The ADH1B*48His allele carriers consume 1,749 g of ethanol per year less (21.8%) than individuals lacking this allele (ADH1B*48Arg/Arg genotype). The differences in alcohol consumption levels were observed for all age groups; however, the statistical significance is compromised (Fig. 2) either due to the inconsistency of the effect or to the small sample size. Since a similar effect of reduced alcohol consumption levels was previously described for other populations on bigger samples (e.g., for Japanese [Matsuo* et al*., 2006] and for Caucasian groups [Sherva *et al*., 2009; Macgregor *et al*., 2009]), it is reasonable to assume that the effect is genuine and statistical significance for Russian males can also be achieved by increasing sample size. The reduced alcohol consumption determined for Russian ADH1B*48His carriers in the current study is close to the published data for other populations; e.g., alcohol consumption was reduced by 18% for white American ADH1B*48His carriers [Sherva* et al*., 2009]. For white Australians, the similar effect varied between 20 and 50% depending on the absolute individual alcohol consumption level (more pronounced for heavy drinkers) [Macgregor *et al*., 2009]. A similar reduction in the amounts of consumed alcohol for Japanese ADH1B*48His carriers was found to depend on the ALDH2*Glu504Lys background. For the *504Glu/ Glu *genotype (normal acetaldehyde detoxication), the presence of ADH1B*48His was found to reduce alcohol consumption by 7.1%. For the *504Glu/Lys *genotype (impeded acetaldehyde detoxication), this level was reduced by 48.1% [Matsuo *et al*., 2006].

We found it interesting to compare the effect of the educational status of an individual with the “protective effect” of the ADH1B*48His allele. The analysis included data for the entire year prior to the survey. An analysis of the ratio of standardized regression Beta coefficients (Table 4) has revealed that alcohol consumption in ADH1B*48His allele carriers is 1.6-fold lower as compared to the previously described effects of higher education levels.

**Table 4:** Regression analysis of the association between the alcohol consumption level and the ADH1B genotype and education level

Linear regression coefficients	Beta*	SE	B	SE	p-value	
Intercept			12864	1260	0.0000
ADH1B genotype (Arg/His vs. Arg/Arg)	-0.073	0.043	-2113	1255	0.0929
Education level (High vs. Low and Medium)	-0.047	0.043	-971	884	0.2722

Note. Abstainers are excluded; nonbeverage alcohol consumers are excluded. *Regression coefficients for standardized data.

The average alcohol consumption (calculated as pure ethanol) was 813 g (10%) lower per year for men with a degree in higher education as compared to those without a degree. Individuals consuming nonbeverage alcohol were excluded from the analysis. Meanwhile, the carriers of the “protective allele” ADH1B*48His had on average consumed 1,749 g (21.8%) less ethanol per person per year (Table 5). The effects of these factors were similar at the population level. Alcohol consumption was 2.5% lower for the ADH1B*48His allele carriers and 2.8% lower for those with a degree in higher education (Table 5). However, since the results were not statistically significant for the selected groups, we chose to compare our preliminary data with the results obtained for larger population groups.

**Table 5:** Alcohol consumption level in relation to genotype and education level

Mean alcohol consumption (g per person per year)		Differences (D)	Contribution of a factor to lower alcohol consumption in the cohort (I)
ADH1B*Arg48His genotype			
Arg/Arg (469 individuals)	Arg/His (60 individuals)	Arg/His vs. Arg/Arg	
8041	6292	21.8%	2.5%
Education level			
Low and Medium (380 individuals)	High (149 individuals)	High vs. Low+Medium	
8071	7259	10.1%	2.8%

Note. Abstainers are excluded; nonbeverage alcohol consumers are excluded.

**Fig. 3:**
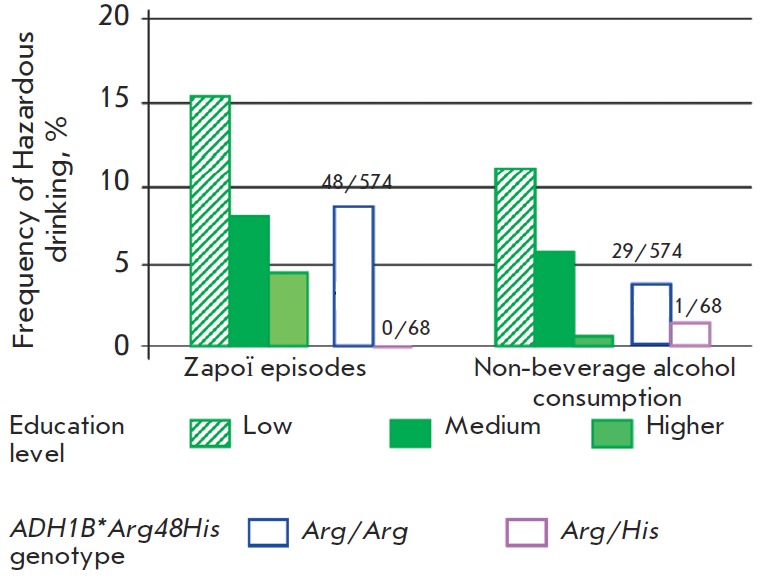
Hazardous drinking in groups with different genotypes and education levels

The effects of genotype on the pattern of alcohol consumption were assessed by comparing the percentage of individuals with heavy drinking problems and individuals involved in the consumption of nonbeverage alcohol among carriers of the ADH1B*48Arg and ADH1B*48His alleles (Fig. 3). No cases of *zapoi *have been detected and only one person was involved in the consumption of nonbeverage alcohol among the group of 68 Russian males, ADH1B*48His allele carriers. On the contrary, these numbers were 8.1% (48 individuals, statistically significant difference, *OR*=12.6, P=0.006) and 5.1% (29 individuals, statistically non-significant), respectively, for the group of 574 ADH1B*48Arg/ Arg genotype carriers. Hence, in this populationbased randomly selected sample of Russian men, the ADH1B*48His allele carriers appear to be protected against *zapoi*. Our data represent the first piece of evidence of the influence of the ADH1B*48His allele on heavy drinking and nonbeverage alcohol consumption behavior among Russian men. A bigger size cohort needs to be studied in order to assess the details of this observation.

The earlier research data involving a group of Russian men of different ethnic backgrounds has indicated that the percentage of individuals with a dangerous pattern of alcohol consumption is lower for individuals with a degree in higher education [2, 21, 27]. The frequency of *zapoi *for Russian men was 5.8% in the higher education subgroup as compared to 8.1% in the secondary and elementary education subgroups.

The higher the education level, the lower the frequency of *zapoi *in the subgroup (Table 6). The result is statistically valid. The conclusion was drawn from an analysis of bigger groups (combined group of 927 men consisting of individuals both with established and not established genotypes). The education level influences the nonbeverage alcohol consumption level as well. The percentage of nonbeverage alcohol consuming persons was higher in the subgroup of individuals without a high education degree (6.0%) as compared to 0.6% in the higher education subgroup (*OR*=10.0, P=0.004) (Fig. 3).

**Table 6:** Association of the incidence of zapoï during the previous year in drinkers and their education level

Did a person have any *zapoï* episodes during the previous year?	Education level (N, %)			Total
	Incomplete secondary	Secondary	Higher
Yes	5 12.20	62 9.31	9 4.09	76 8.20
No	36 87.80	604 90.69	211 95.91	851 91.80
Total	41 100.00	666 100.00	220 100.00	927 100.00

Note. Pearson χ2 = 6.8939 Pr = 0.032.

The comparison of the “protective effects” of higher education and presence of the ADH1B*48His allele indicates that “genetic protection” is more effective at the individual level, whereas both factors are equally effective at the group level, (Tables 4 and 5).

It has been demonstrated that the ADH1B*48His allele is associated with a lower risk of incidence of *zapoi* in Russian males and with lower overall alcohol consumption on average by 1,749 g (2186 ml) of pure ethanol per person per year. This effect is 2–3 times lower than that of the “dry law” campaign practiced in the late 1980s, which had lowered alcohol consumption to 4–6 L per individual per year [28, 29]. The restriction of alcohol sales has an effect on the entire population. The established protective effect of the ADH1B*48His allele corresponds only to 10% of Russian males. However, the frequency and significance of this allele may be higher in other ethnic groups of the Russian Federation. Hence, research into the effects of the ADH1B*48His allele on alcohol consumption in different ethnic groups in Russia may be important.
